# Protocol for synthesizing and purifying short-length poly(ADP)ribose polymer using fast protein liquid chromatography

**DOI:** 10.1016/j.xpro.2025.104319

**Published:** 2026-01-12

**Authors:** Singh Neeharika, Dagur Singh Hanuman, Eerappa Rajakumara

**Affiliations:** 1 Macromolecular Structural Biology, Department of Biotechnology, Indian Institute of Technology, Hyderabad, Telangana 502285, India

**Keywords:** cancer, molecular biology, protein biochemistry

## Abstract

Here, we present a protocol for synthesizing and purifying high-yield, short-length poly(ADP)ribose (PAR) polymers using fast protein liquid chromatography (FPLC). We describe steps for expressing and purifying proteins, *in vitro* synthesis of PAR, and fractionation and visualization of PAR. This cost-effective, non-hazardous, shorter approach avoids the use of radiolabeled substrates, hazardous reagents, and specialized equipment, producing homogenous PAR chains under 10 units. It eliminates the need for enzymes such as PARG and SVP, enabling broad accessibility for biophysical and structural studies.

## Highlights

• Steps for purifying human PARP1 through immobilized metal affinity chromatography
• Procedure for synthesis of poly(ADP)ribose
• Guidance on fractionation of poly(ADP)ribose using Resource Q column
• Instructions for purification of short-length PAR

## Abstract (editor highlights)

Publisher’s note: Undertaking any experimental protocol requires adherence to local institutional guidelines for laboratory safety and ethics.

## Graphical abstract

**Figure:**
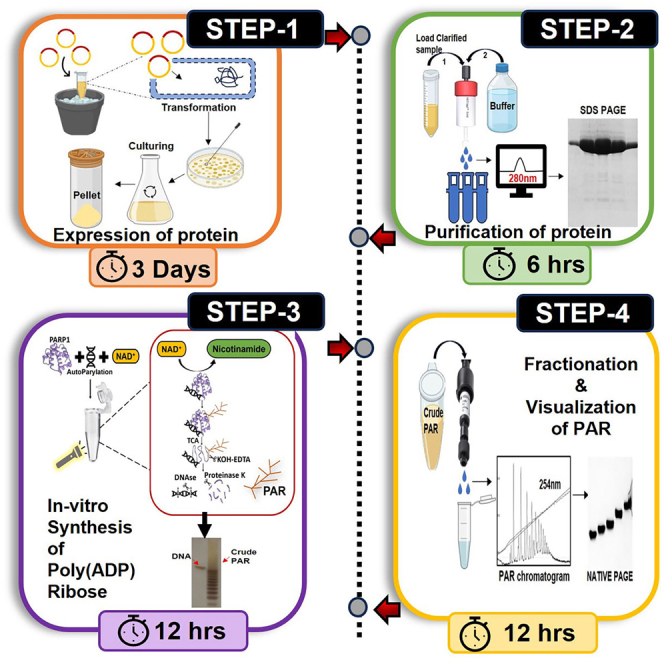

## Before you begin

Poly(ADP)ribose (PAR),^1^^,^^2^ synthesized by PARP family enzymes, on themselves (self-PARylation) and on hundreds of other proteins, DNA, RNA, etc., using NAD^+^ as a substrate,^3^^,^^4^ regulates critical cellular processes including DNA damage repair, gene expression, chromatin remodeling, and cell death.^4^^,^^5^^,^^6^^,^^7^^,^^8^ Among the 17 PARP enzymes,^4^^,^^9^ PARP1 is the most abundant and well-studied, rapidly producing PAR chains in response to DNA damage.^3^^,^^10^ Its activity results in PAR chains ranging from 2 to over 244 units,^11^ which can be linear or branched.^12^ Chain length plays a key role in the function and binding specificity of PAR.^12^^,^^13^ Specific proteins preferentially bind short PAR chains, while others favor longer ones, impacting diverse signaling pathways.^12^^,^^13^^,^^14^ However, a significant barrier to structural and biophysical analysis of PAR, protein interactions is the lack of scalable, efficient, and safe protocols for generating high-purity PAR polymers of defined lengths.^15^^,^^16^ Most of the studies for PAR synthesis typically relied on radiolabeled NAD^+^, histones, HPLC purification, and enzymatic digestion (e.g., with PARG or SVP), making them expensive, hazardous, and inefficient for large-scale use.^17^^,^^18^ Chemical synthesis approaches remain limited to mono or di-ADPr and require specialized expertise and equipment.^19^

### Innovation

To overcome above limitations, we developed a cost-effective, non-hazardous approach for the large-scale in vitro synthesis and purification of short-length PAR polymers. This protocol uses recombinant PARP1 stimulated by 16-mer DSB DNA, and purification via Resource Q column on FPLC, without ethanol precipitation or radiolabeling. Additional cost-effective advantages of this protocol are: (A) FPLC equipment is used for both recombinant PARP1 and any other proteins purification and PAR fractionation instead of HPLC equipment which performs under high-pressure and unsuitable for protein purification (B) Resource Q column used in this protocol is suitable for both proteins and nucleic acid purification using FPLC instead of C18 or DNA Pac PA100 which are compatible with only HPLC. Therefore, this protocol doesn’t require two different chromatography systems and columns for purifying PAR synthesizing enzyme, PARP1, and PAR fractionation. Native PAGE analysis confirms a homogenous PAR product suitable for structural and biophysical assays. This approach yields approximately 600–300 nmol of 6–12-mer PAR species, and 250–100 nmol of 13–20-mer species per length and per batch. The overall yield is about 15%–20% per batch, which is sufficient for biophysical and biochemical studies. The protocol describes the specific steps for synthesizing and purifying short-length PAR polymers using recombinant human PARP1 and FPLC purification.

## Key resources table

**Table:** 

REAGENT or RESOURCE	SOURCE	IDENTIFIER
**Bacterial and virus strains**		

*E. coli* Rosetta2 (DE3)	Merck	Cat#71397-3CN

**Chemicals, peptides, and recombinant proteins**		

Luria Bertani (LB) Broth	HiMedia	Cat#M1245-500G
Isopropyl β-D-1-thiogalactopyranoside (IPTG)	SRL	Cat#54110
Chloramphenicol	VWR Life Sciences	Cat#0230-100G
Kanamycin	SRL	Cat#99311
Nickel (II) Sulphate Hexahydrate	HiMedia	Cat#10101970
Imidazole	HiMedia	Cat#288324
Acrylamide	HiMedia	Cat#79061
β-Mercaptoethanol	HiMedia	Cat#60242
Glycerol	HiMedia	Cat#56815
Bis-acrylamide	HiMedia	Cat#110269
Tris Hydrochloride (Tris HCl)	HiMedia	Cat#1185531
Tris Base	HiMedia	Cat#77861
Ammonium Per Sulphate (APS)	SRL	Cat#7727540
Nicotinamide Adenine Dinucleotide (NAD^+^)	HiMedia	Cat#53849
Trichloroacetic acid (TCA)	HiMedia	Cat#76039
Sodium Dodecyl Sulphate (SDS)	SRL	Cat#151213
Potassium Hydroxide (KOH)	Sigma	Cat#1310583
Ethylenediaminetetraacetic acid (EDTA)	HiMedia	Cat#6381926
Phenol	SRL	Cat#108952
Chloroform	SRL	Cat#67663
Isoamyl alcohol	HiMedia	Cat#123513
Ethanol	Hayman	Cat#F204325
Phenylmethylsulfonyl fluoride (PMSF)	HiMedia	Cat#329986
EDTA-free Protease Inhibitor Cocktail	Roche	Cat#11836170001
Lysozyme	SRL	Cat#12650883
Proteinase K	MagGenome	Cat#MG19PK-1
Silver Nitrate	SRL	Cat#7761888
Sodium Carbonate Anhydrous	HiMedia	Cat#497198
Magnesium Chloride Hexahydrate (MgCl2)	HiMedia	Cat#7791186
Acetic Acid	HiMedia	Cat$64197
Formaldehyde	Sigma	Cat#605001005
Nitric acid	Sigma	Cat#1004410250
Unstained Protein Marker	Thermo Scientific	Cat#26610

**Oligonucleotides (5′-3′)**		

16-mer DSB DNA (5′–3′) Strand 1: GACGACCCGGAGCACA Strand 2: TGTGCTCCGGGTCGTC	Huwel Lifesciences	Custom synthesis
10mer-ssDNA: 5′-CTAGAAGATG-3′		

**Recombinant DNA**		

pRSF-Duet1-PARP1 Expression Vector	GenScript	Custom synthesis

**Other**		

pH Strips	Sigma	Cat#109543
Millex Nylon syringe filter (0.45 μm)	Sigma	Cat#SLHN033
Vivaspin 2-kDa Centrifugal Concentrator	Sartorius	Cat#VN01H91
5 mL HiTrap IMAC HP column	Cytiva	Cat#17524701
1 mL Resource Q column	Cytiva	Cat#17117901
15 mL Amicon Ultra Centrifugal Filter (30 kDa)	Sigma	Cat#UFC903008

## Materials and equipment

Silver staining can be performed as reported earlier.^20^^,^^21^ Care should be given to dissolve the PAR pellet obtained after TCA precipitation, which can be achieved with sonication and manual pipetting. Extensive centrifugation using a Vivaspin column is not recommended, and the column should be discarded after 3–5 uses. The OD at 258 nm wavelength is recorded using the NP80 Touch Nano Photometer (Implen, Germany).

**Table:** Lysis Buffer

Reagents (stock concentration)	Final concentration	Amount (for 1000 mL)
Tris HCl pH 8.0, (1 M)	50 mM	50 mL
Imidazole (4 M)	30 mM	7.5 mL
NaCl (5 M)	500 mM	100 mL
PMSF (100 mM)	1 mM	10 mL
Glycerol (100%)	10%	100 mL
β-Mercaptoethanol (14.3 M)	3 mM	209 μL
Lysozyme (50 mg/mL)	0.1 mg/mL	2 mL
Protease Inhibitor (50 Tablets)	1 Tablet/purification	1 Tablet
Total	N/A	–

**Table:** Stripping Buffer

Tris HCl pH 7.5, (1M)	50 mM	50 mL
NaCl (5M)	500 mM	100 mL
EDTA pH 7.5 (1M)	50 mM	50 mL
Total	N/A	–

**Table:** IMAC Buffer A

Tris HCl pH 8.0, (1 M)	50 mM	50 mL
Imidazole (4 M)	30 mM	7.5 mL
NaCl (5 M)	500 mM	100 mL
β-Mercaptoethanol (14.3 M)	3 mM	209 μL
Glycerol (100%)	10%	100 mL
Total	N/A	–

**Table:** IMAC Buffer B

Tris HCl pH 8.0, (1 M)	50 mM	50 mL
Imidazole (4 M)	75 mM	18.75 mL
NaCl (5 M)	500 mM	100 mL
β-Mercaptoethanol (14.3 M)	3 mM	209 μL
Glycerol (100%)	10%	100 mL
Total	N/A	–

**Table:** IMAC Buffer C

Tris HCl pH 8.0, (1 M)	50 mM	50 mL
Imidazole (4 M)	500 mM	125 mL
NaCl (5 M)	500 mM	100 mL
β-Mercaptoethanol (14.3 M)	3 mM	209 μL
Glycerol (100%)	10%	100 mL
Total	N/A	–

**Table:** PARP1 Buffer D

Reagents (stock concentration)	Final concentration	Amount (for 50 mL)
Tris HCl pH 8.0 (1 M)	50 mM	2.5 mL
NaCl (5 M)	300 mM	3 mL
β-Mercaptoethanol (14.3 M)	2 mM	7 μL
Glycerol (100%)	10%	5 mL
Total	N/A	–

**Table:** Automodification (AM) Buffer

Tris HCl pH 8.0, (1 M)	50 mM	2.5 mL
NaCl (5 M)	100 mM	1 mL
MgCl_2_ (1 M)	10 mM	0.5 mL
Total	N/A	–

**Table:** Buffer E

Reagents (stock concentration)	Final concentration	Amount (for 10 mL)
Tris HCl pH 8.0 (1 M)	50 mM	0.5 mL
NaCl (1 M)	10 mM	0.1 mL
MgCl_2_ (1 M)	10 mM	0.1 mL
CaCl_2_ (1 M)	5 mM	0.05 mL
Total	N/A	–

**Table:** Buffer F

Reagents (stock concentration)	Final concentration	Amount (for 250 mL)
Tris HCl pH 8.0 (1 M)	50 mM	12. 5 mL
NaCl (5 M)	10 mM	0.5 mL
Total	N/A	–

**Table:** Buffer G

Tris HCl pH 8.0 (1 M)	50 mM	12. 5 mL
NaCl (5 M)	1000 mM	50 mL
Total	N/A	–

## Step-by-step method details

### Recombinant poly(ADP)ribose polymerase 1 expression

**Timing: 3 days**

This section describes the expression of recombinant full-length human PARP1 in bacterial cells.

1. Bacterial Expression of human PARP1.
  a. **On day 1**, take out the *E. coli* Rosetta 2 (DE3) competent cells from the −80°C freezer and thaw them on ice for 15 min.
  b. Add 100 ng of plasmid DNA encoding full-length PARP1 to 50 μL of competent cells and further incubate on ice for 30 min.
  c. Perform heat shock at 42°C for 90 s on a dry bath, followed by immediate cooling on ice for 4–5 min.
  d. Add 1 mL of LB medium (freshly prepared) and incubate in the bacterial incubator shaker at 37°C at 180 rpm speed for 1 h.
  e. Plate the cells onto prewarmed LB Agar plates containing kanamycin (50 μg/mL) and chloramphenicol (30 μg/mL). Keep the plates for 12–14 h at 37°C in a bacterial incubator.
2. **On day 2**, pick a single transformed colony in 60 mL LB broth medium supplemented with 50 μg/mL kanamycin and 30 μg/mL chloramphenicol and incubate it at 37°C for 12–16 h at 180 rpm in a bacterial incubator shaker.
3. **Next day,** distribute 10 mL pre-culture into six flasks, each filled with 1000 mL autoclaved LB media.
  ***Note:*** Autoclave LB media at 121°C and 15 psi for 15 min.
  a. Add 50 μg/mL kanamycin and 30 μg/mL chloramphenicol.
  b. Measure OD at 600 nm after 5–6 h.
  c. Upon reaching an optical density at 600 nm (OD_600_) of approximately 0.9, add 0.5 mM IPTG supplemented with 10 mM benzamide and further incubate this large-scale culture at 20°C for 24 h.
    ***Note:*** Benzamide has modest affinity for PARP1. It may mitigate the nature of PARP1. So, adding benzamide at this stage is primarily precautionary and may provide slight stabilization to the catalytic domain of PARP1.
  d. Harvest cultured cells by centrifugation at 6000 *g* for 15 min at 20°C.
    **CRITICAL:** Six liters of culture are required for enough recombinant PARP1. The average weight of the pellet obtained from 6 L was 30 ± 2 g**.**

### Purification of PARP1 through immobilized metal affinity chromatography

**Timing: 6 h**

This section describes the purification of recombinant full-length human PARP1.

**Figure 1:**
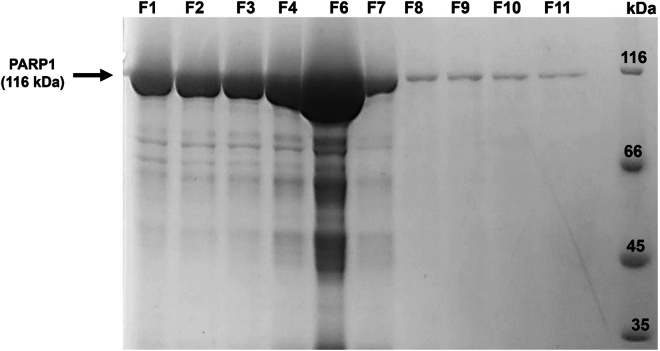
Representative SDS-PAGE image from the purification of Full-length Poly(ADP)ribose polymerase (PARP1) Purification of PARP1 by IMAC. PARP1 was eluted at 250 and 500-mM imidazole concentrations with 70% pure samples. Fractions F1-F4 and F6-F11 represent the elution at 250 mM and 500 mM imidazole, respectively. We pooled pure PARP1 samples (F1-F4 and F6-F10) and concentrated them using an Amicon Centrifugal Filter (30-kDa) for further use as described in the protocol.

4. Resuspend the cell pellet in lysis buffer.
  a. Sonicate the resuspended cell lysate in a 50 mL Falcon tube, keeping the sample on ice.
    ***Note:*** Sonicator settings are as follows: 5 s “ON”, 20 s “OFF”, 50%–60% Amplitude, six cycles each of 2.5 min.
    ***Note:*** Sonicator type and model: Qsonica Q125 (USA), with maximum power output of 125 W operated at 25 kHz, equipped with a probe of 6.4 mm tip diameter.
  b. Clarify the sonicated sample by centrifugation at 20,000 g for 30 min at 4°C.
  c. Connect the HisTrap column (5 mL volume) to the Fast Protein Liquid Chromatography (FPLC) system.
5. Recharge the HisTrap column (5 mL Column Volume (CV)). All steps are performed at a Flow Rate of 3 mL/min.
  a. Pass 2 CV of water and 3 CV of stripping buffer through the column.
  b. Equilibrate the column with 4 CV of IMAC Buffer A and further rinse the column with 2 CV of water.
  c. Charge the column with 2 CV of 0.2 M NiSO_4_ and further.
  d. Wash with 3 CV of water.
  e. Re-equilibrate with 2 CV of IMAC Buffer A.
6. Load clarified supernatant onto the HisTrap column. (**Note**: Flow rate is 3 mL/min in subsequent steps).
  a. Pass 6 CV of IMAC buffer A.
  b. Wash the column with 10 CV of IMAC buffer B.
  c. Prepare a gradient using IMAC buffer B and IMAC buffer C by manually increasing the percentage of IMAC buffer C in the system (6%, 12%, 24%, 48% and 100% buffer C) and collecting the eluting protein fractions. Analyze the purity of eluted fractions on 12% SDS-PAGE (Figure 1).
  d. Collect eluted PARP1 fractions and concentrate them to a 1 mL volume using an Amicon centrifugal filter unit (cut-off∼30-kDa).
    ***Note:*** Fractions showing a prominent band at the expected molecular weight on SDS–PAGE were identified as the target protein and pooled. Typically, up to 30%–40% impurity is considered tolerable at this stage.
  e. Perform buffer exchange using a 30-kDa Amicon centrifugal filter by repeatedly concentrating the protein to 1 mL and diluting with an equal volume of PARP1 Buffer D thrice, allowing efficient replacement of the original buffer while retaining the protein.
    ***Note:*** The Ni-NTA purified full-length PARP1 (∼70% pure) is enzymatically active and capable of producing PAR. Choosing the desalting step of Ni-NTA purified PARP1 over gel filtration prevents the loss of PARP1 yield. Also, PARP1 is highly prone to degradation.
  f. Further concentrate the protein samples to 10 mg/mL or ∼86.2 μM using a 30-kDa Amicon Centrifugal Filter, and the concentration was determined by measuring absorbance at 280 nm using an Implen Nanophotometer.
    ***Note:*** The PARP1 is now in buffer D at a concentration of 10 mg/mL or 86.2 μM.
    **CRITICAL:** PARP1 is a multidomain protein and less stable at 25°C. It is highly prone to degradation, so purification should be performed at a 4°C cold chamber or the PARP1 fractions should be kept on ice.
    ***Note:*** Storage conditions of buffers in this step are 4°C.

### *In vitro* synthesis of poly(ADP)ribose

**Timing: 12 h**

PARP1 is known to produce PAR polymer in the presence of damaged DNA and nicotinamide adenine dinucleotide (NAD^+^) as a cofactor. This section describes the synthesis of crude PAR.

7. Set up 20 individual ADP-ribosylation reactions, each with a total volume of 1 mL, in separate 1.5 mL Eppendorf vials.
  a. For each reaction, incubate PARP1 (10 μM) with 16-mer double-stranded break DNA (hereafter called DSB) at a final concentration of 100 nM in automodification buffer (AMB) on ice for 60 min.
  b. Prepare a 100 mM NAD^+^ stock solution in autoclaved filtered water. Add 2.5 mM NAD^+^ (25 μL) from 100 mM stock to each reaction vial and incubate the reaction at 25°C for 1.5 h.
  c. Stop the reaction by adding 220 μL of 100% Trichloroacetic Acid (TCA) to achieve a final concentration of 20%, mix well, and centrifuge for 15 min at 4°C at 15000 g.

***Note:*** TCA rapidly terminates ADP-ribosylation by efficiently precipitating proteins.

8. Decant the supernatant.
9. Prepare 5% TCA stock and keep it on ice for 30 min. Add 1 mL volume of this prechilled TCA to each vial, and centrifuge for 15 min at 4°C at 15000 g, then remove the supernatant carefully without disturbing the pellet.
10. Add 1 mL volume of pre-cooled absolute ethanol, and centrifuge for 15 min at 4°C at 15000 g and decant the supernatant.
11. Heat all samples at 55°C for 2 h to remove residual ethanol.
12. Collect the pellet and scrap all whitish material attached to the walls of the vials using 0.2–10 μL microtips to ensure maximum recovery and pool them in one 1.5 mL vial.
  ***Note:*** In step 11, all residual ethanol must be removed from the vial, as scratching the whitish material in step 12 will be easier. Alternatively, samples can be kept at 37°C for 12–14 h (**under step 11).** Temperatures above 55°C, are avoided, as higher temperatures degrade poly(ADP)ribose.
  **CRITICAL:** Care was taken to thoroughly scrape the entire layer on the vial walls along with the pellet to maximize recovery of Poly (ADP)ribose product and minimize its loss.
  a. Add 500 μL buffer E to the pellet, dissolve the pellet and perform sonication for 2 min (Pulses: 10s ‘ON’ and 30s ‘OFF’, 20% Amplitude) using an immersion probe of 5/64′′ (2 mm) diameter.
    ***Note:*** Make sure the probe is not contaminated. If the pellet is not dissolved in 2–3 cycles, decrease the amplitude to 5% and increase the number of cycles to 5–6.
  b. Add DNase I (10 Units) and incubate the sample for 30 min at 37°C.
  c. Add SDS and Proteinase K to the sample to reach a final concentration of 0.2% (10% stock) and 0.5 mg (20 mg stock), respectively, and keep it at 50°C for 3 h.
  d. Now, add an equal volume of 100 mM KOH and 5 mM EDTA and heat the sample for 2 h at 60°C to detach the PAR polymers from PARP1.
  e. Adjust the pH to 8.0 using concentrated HCl.
    **CRITICAL:** Solution should have pH 8.0, and due to the small volume, the pH is checked using litmus paper.

Before proceeding, prepare a PCI solution using Phenol: Chloroform: Isoamyl alcohol (PCI) at a ratio of 25:24:1 in a 1 mL volume.

13. Add an equal volume of PCI solution and vortex it for 1 min.
14. Centrifuge the sample at 13500 g for 10 min at 25°C.
15. Collect the aqueous layer and mix it with an equal volume of chloroform and vortex for 1 min.
16. Centrifuge at 13500 g for 10 min at 25°C.
17. Repeat the above step one more time.

**CRITICAL:** Carefully collect the aqueous layer and avoid collecting the organic layer. Filter the collected aqueous layer using syringe filter paper (pore size 0.45 μm).

### Purification, fractionation, and visualization of PAR polymer

**Timing: 12 h**

This step describes the fractionation of crude PAR and the purification of a single PAR polymer using ion-exchange chromatography.

**Figure 2:**
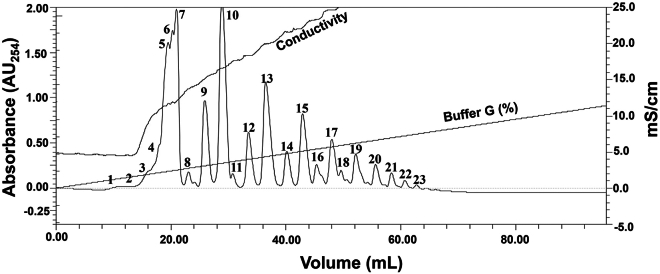
Chromatogram of FPLC-based fractionation of poly(ADP)ribose (PAR) polymers PAR polymers were separated using anion exchange chromatography on a Resource Q column. Elution was carried out using a linear salt gradient from 0%–100% Buffer G (Buffer F: 10 mM NaCl, 50 mM Tris HCl pH 8.0; Buffer G: 1 M NaCl, 50 mM Tris HCl pH 8.0) at a flow rate of 0.25 mL/min. The UV absorbance at 254 nm is plotted on the left Y-axis, while the conductivity is shown on the right Y-axis. The percentage of Buffer G over time is marked. Peak fractions, marked numerically on the chromatogram, were collected and further analyzed by native PAGE and silver staining (see Figure 3). The X-axis represents the elution volume in millilitres (mL).

18. Add 2.5 mL buffer F to the collected aqueous layer.
19. Connect the Resource Q column to FPLC. Set the wavelength to 254 nm in the FPLC UV-detector system.
  a. Pass 3 mL autoclaved water with a 0.2 mL/min flow rate. (Note: Flow rate is 0.2 mL/min in subsequent steps).
  b. Equilibrate the column with 5 mL buffer F.
  c. Load the PAR sample onto the column.
  d. Pass 10 mL buffer F.
20. Run a gradient buffer F (100%–0%) and Buffer G (0 - 100%) for a total of 200 mL volume.
21. Collect each eluted peak manually into a separate 1.5 mL vial until the final peak appears on the chromatogram (Figure 2), ensuring that each fraction is collected individually without overlap (Figure 2).
22. Keep all eluted samples in the vacuum evaporator for 4 h (two cycles, each of 2 h) to minimize the volume.
23. Prepare 12 μL volume of each eluted and vacuum evaporated sample (take 1–3 μL volume of highly concentrated, prominent peaks, and 4–9 μL volume for medium to small peaks).
24. Prepare the 10-mer single-stranded DNA (ssDNA) (100 pmol) and 16-mer duplex DNA (100 pmol) for a control to know the average size of eluted PAR polymer samples.

**Figure 3:**
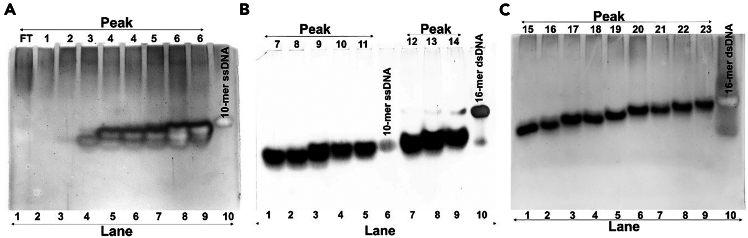
Analysis of FPLC-fractionated PAR polymer on native PAGE (A–C) Native PAGE followed by silver staining was used to analyze individual fractions of synthesized poly(ADP)ribose (PAR) polymers without precipitating with ethanol and sodium acetate, and separated using anion exchange FPLC (Resource Q column). Peak numbers are indicated above each panel, and the corresponding lane numbers are marked below. (A) PAR fractions corresponding to Peaks 1–6 are shown. Lane 1 contains the flow-through (FT). Lane 10 is a control 10-mer single-stranded DNA (ssDNA). (B) PAR fractions corresponding to Peaks 7–11 and 12–14 are shown. Lane 6 contains a 10-mer ssDNA control, and Lane 10 contains a 16-mer double-stranded break (DSB) DNA control (C) PAR fractions corresponding to Peaks 15–23 are shown. Lane 10 contains the 16-mer DSB DNA control.

Run all samples on 20% native PAGE (Figures 3A–3C).

***Note:*** All samples, including controls, were mixed with 1× DNA loading dye (6× stock solution) in 12 μL volume. Volume was adjusted using buffer F for highly concentrated samples or controls. This step can be repeated with different volumes if no bands are found on the gel**)**.

***Note:*** Run at 80 V for 90 min or till ¾ of the gel.

25. Perform Silver staining to visualize each PAR sample.

***Note:*** Use standard protocol for silver staining. (See under ref.^20^^,^^21^).

26. Take the desired PAR peak fraction and concentrate it to 0.3 mL volume using the Vivaspin centrifugal filter unit (2-kDa, 0.5–6 mL volume) at speed 7000 g for 10 min.
  ***Note:*** If the collected volume is high, repeat the process for 5–6 cycles to achieve the final 0.3 mL volume. This step can be omitted if the final volume of the PAR fraction is 0.2 to 0.3 mL.
  a. Add 0.3 mL autoclave-filtered water to the PAR fraction in the Vivaspin centrifugal filter unit and again centrifuge it to 0.3 mL volume.
    ***Note:*** Repeat this step two more times.
  b. In the last step, further concentrate the PAR sample to a 30–150 μl volume.
    ***Note:*** Volume may vary depending on the user’s requirement.
  c. Prepare a 1000–2000 times dilution and measure the OD at 258 nm in the Nanophotometer (Implen, Germany).
  d. Determine concentration using the extinction coefficient of ADPr (13500 M^–1^ cm^–1^).
    ***Alternatives:*** Any other spectrophotometer can also be used to measure the OD at 258 nm.

## Expected outcomes

Upon completing this protocol, users should obtain a high yield of short-length poly(ADP)ribose (PAR) polymers from recombinant PARP1, suitable for biophysical and structural studies. TCA precipitation followed by ethanol washing and sonication ensures thorough dissolution of the pellet, eliminating the need for ethanol and sodium acetate precipitation in the later steps, which prevents the loss of short PAR chains. Direct purification using a Resource Q column via FPLC yields distinguishable peaks (Figure 2), corresponding to PAR fractions of various lengths (Figure 3). Visualization on native PAGE confirms that fractions 1–11 migrate below a 10-mer ssDNA marker (Figures 3A and 3B), indicating successful enrichment of short PAR species (Figures 3A and 3B). Longer PARs were also eluted, but with less quantity (Figure 3C). Each collected fraction can be concentrated and buffer-exchanged efficiently using centrifugal filters (MWCO ∼2 kDa), with a final yield exceeding 50% from an initial 37.5 μmol NAD^+^ reaction, sufficient for downstream biophysical and structural applications. Notably, the protocol does not require radiolabeled NAD^+^, histone substrates, PARG or SVP enzymes, or HPLC systems, making it a cost-effective, non-hazardous, and accessible method for laboratories seeking a simplified approach to obtain short-length PAR polymers in high yield.

## Quantification and statistical analysis

Prepare dilutions of each concentrated fraction and measure the concentration of polymer at 258 nm using a Nanophotometer (Implen, Germany). We use the ADPr extinction coefficient to estimate the final concentration of the PAR polymer. (**See:** sub-step d under step 26).

## Limitations

As is evident, NAD^+^ controls the polymer size produced by PARP1.^15^ We only used a limited amount of NAD^+^ to produce short-length PAR, but to produce longer-length PAR, the amount of NAD^+^ can be increased, and our described protocol can be applied to get a large amount of longer-length PAR polymer. Mass-spectrometry has not been performed, which can tell the exact size of the PAR polymer; instead, we use custom-synthesized single-stranded and double-stranded DNA oligonucleotides, while the use of mass-spectrometry makes the whole process costly. If any user has access to a mass spectrometer, they can use it to determine the exact mass of the desired PAR polymer. Other types of chromatography methods were not tested in the described study, which could also yield an adequate amount of PAR polymer. We use a 1 mL resource Q column, which is sufficient to hold an adequate amount of PAR polymer, but other volume types can also be tested.

## Troubleshooting

### Problem 1

Low expression of Full-length human PARP1.

### Potential solution

Gene construct in the pET28 expression vector could result in low expression of PARP1. In that case, use 1% glucose during primary culture, and add 5–10 mM benzamide in secondary culture. User, make sure to add IPTG when OD reaches 0.9 to 1.0.

### Problem 2

PAR pellet (debris obtained in step 12) is not dissolved properly.

### Potential solution

Perform sonication, but ensure that the probe of the Sonicator is not contaminated. If the pellet is not dissolved in 2–3 cycles, decrease the amplitude to 5% and increase the number of cycles to 5–6.

### Problem 3

The peaks obtained in the chromatograms are not well separated.

### Potential solution

This could be due to the large amount of PAR polymer loaded onto the resource Q column. Decrease the amount of PAR on the column.

### Problem 4

If no peaks appeared, or abnormal PAR peaks appeared with unusual bumps in the NaCl gradient in step 20.

### Potential solution

No peaks indicate that the user has no experience in chromatography. Abnormal PAR peaks with unusual bumps in the NaCl gradient indicate that air bubbles or particles have entered the column or pump connected to the FPLC. In that case, dilute the PAR aqueous layer in Buffer F to 5–6 mL, then filter it with a syringe filter. Make sure the syringe filter is not damaged.

## Resource availability

### Lead contact

Further information and requests for resources should be directed to and will be fulfilled by the lead contact, Eerappa Rajakumara (eraj@bt.iith.ac.in).

### Technical contact

Technical questions on executing this protocol should be directed to and will be answered by the technical contacts, Dagur Singh Hanuman (bo20m21p100001@iith.ac.in) and Singh Neeharika (bt23resch11009@iith.ac.in).

### Materials availability

All enzymes and reagents used in the protocol are commercially available.

### Data and code availability

No data and codes were generated in this study.

## Acknowledgments

S.N. thanks the Ministry of Education (MoE) and the Indian Institute of Technology, Hyderabad (IITH), for the fellowship. D.S.H. thanks the Prime Minister’s Research Fellowship (MoE) for the fellowship.The authors thank the Department of Biotechnology, IITH, and the Ministry of Science & Technology, Department of Biotechnology (DBT), India, for providing the resources and facilities used in this study.

## Author contributions

S.N. and D.S.H. wrote the initial draft. E.R. conceived the study. S.N. and D.S.H. performed all the experiments in the steps described in the protocol. All authors contributed equally to the final version of the manuscript.

## Declaration of interests

The authors declare no competing interests.
